# An Integrated Framework for Assessing Vulnerability to Climate Change and Developing Adaptation Strategies for Coffee Growing Families in Mesoamerica

**DOI:** 10.1371/journal.pone.0088463

**Published:** 2014-02-26

**Authors:** María Baca, Peter Läderach, Jeremy Haggar, Götz Schroth, Oriana Ovalle

**Affiliations:** 1 International Center for Tropical Agriculture (CIAT), Managua, Nicaragua; 2 Natural Resources Institute (NRI), University of Greenwich, Chatham Maritime, Kent, United Kingdom; 3 Rainforest Alliance, Wageningen, The Netherlands; 4 International Center for Tropical Agriculture (CIAT), Cali, Colombia; DOE Pacific Northwest National Laboratory, United States of America

## Abstract

The Mesoamerican region is considered to be one of the areas in the world most vulnerable to climate change. We developed a framework for quantifying the vulnerability of the livelihoods of coffee growers in Mesoamerica at regional and local levels and identify adaptation strategies. Following the Intergovernmental Panel on Climate Change (IPCC) concepts, vulnerability was defined as the combination of exposure, sensitivity and adaptive capacity. To quantify exposure, changes in the climatic suitability for coffee and other crops were predicted through niche modelling based on historical climate data and locations of coffee growing areas from Mexico, Guatemala, El Salvador and Nicaragua. Future climate projections were generated from 19 Global Circulation Models. Focus groups were used to identify nine indicators of sensitivity and eleven indicators of adaptive capacity, which were evaluated through semi-structured interviews with 558 coffee producers. Exposure, sensitivity and adaptive capacity were then condensed into an index of vulnerability, and adaptation strategies were identified in participatory workshops. Models predict that all target countries will experience a decrease in climatic suitability for growing Arabica coffee, with highest suitability loss for El Salvador and lowest loss for Mexico. High vulnerability resulted from loss in climatic suitability for coffee production and high sensitivity through variability of yields and out-migration of the work force. This was combined with low adaptation capacity as evidenced by poor post harvest infrastructure and in some cases poor access to credit and low levels of social organization. Nevertheless, the specific contributors to vulnerability varied strongly among countries, municipalities and families making general trends difficult to identify. Flexible strategies for adaption are therefore needed. Families need the support of government and institutions specialized in impacts of climate change and strengthening of farmer organizations to enable the adjustment of adaptation strategies to local needs and conditions.

## Introduction

Climate change represents a serious threat for Mesoamerican countries due to the multiple impacts predicted to directly affect the population as well as various sectors of the economy [1], [2]. The Vulnerability-Resilience Indicators developed by Yohe suggested high exposure to climate change for the Mesoamerican and Caribbean Region [14]. Climate projections indicate that increases in temperature will reduce crop yields in general [3] and particularly those of Arabica coffee, one of the region's major exports. Arabica coffee responds strongly to seasonal temperature patterns and coffee of high quality requires relatively stable temperatures within a fairly narrow range [4], [5]. Läderach and others [6] predicted that optimal conditions for growing Arabica coffee in Mesoamerica will move from currently 800 to 1,400 m.a.s.l upwards to 1,200 to 1,600 m.a.s.l by 2050. Studies in Ethiopia and Kenya have similarly foreseen significant impacts of climate change on the distribution of wild coffee and coffee pests [7], [8], in the later case extending the areas affected by coffee berry-borer.

During the last 40 years, agriculture has contributed 10% of the GDP in Latin American countries and is a major export earner. It is an important sector in the regional economy, since it employs 30% to 40% of the economically active population and is essential for the food security of the poorest segment of society [3], [9]. Across Mexico and Central America, over 4 million people depend directly on coffee production for their livelihoods [10]. According to CEPAL [9], coffee production, purchasing, and processing employ an estimated 8.5 million people in the region. Employment and income generation from coffee are particularly significant for many indigenous peoples in Mexico and Guatemala. The environmental services generated by shade coffee farms, including carbon sequestration, watershed services and the conservation of biodiversity, have also been highlighted by many authors [11], [12], while Haggar and others [13] have shown how land-use change can affect the provision of environmental services when shade coffee is replaced by other land-uses [13].

The Intergovernmental Panel on Climate Change (IPCC) presents an integrated concept where “vulnerability to climate change is the degree by which a system is susceptible or unable to face the adverse effects of climate change, including climate extremes and variability. Moreover, vulnerability depends on the nature, magnitude, and rate of climate change, as well as the variation to which a system is exposed, its sensitivity and its capacity for adaptation”. Exposure is the nature and extent of changes that a place’s climate is subjected to with regard to variables such as temperature, precipitation, and extreme weather events. Sensitivity is a measure of how systems could be affected by the change in climate (e.g. how much crop yields change or how much human health might be affected). In contrast, adaptive capacity is defined as a system’s ability to adjust to climate change in order to reduce or mitigate possible damage [3]. Adaptive capacity is dynamic, and depends partly on the societý productive base, such as: natural and artificial assets, social benefits and networks, human capital and institutions, governance, national income, health and technology [2], and how much capability a society has to adapt to the changes so as to maintain, minimize loss of, or maximize gain in welfare.

The current study was conducted to evaluate the vulnerability of coffee farming communities in El Salvador, Guatemala, Mexico and Nicaragua and to identify adaptation strategies to climate change [1], [3].

## Materials and Methods

In order to assess the vulnerability to climate change and define appropriate adaptation strategies, we adapted the IPCC's definition of vulnerability and applied it to small coffee producers [3]. For our methodology, vulnerability is defined as changes in climate variables that affect agricultural and natural systems over a timeframe. The vulnerability in the livelihoods of small coffee farmers is a function of three factors: exposure, sensitivity and adaptive capacity.

**Figure 1:**
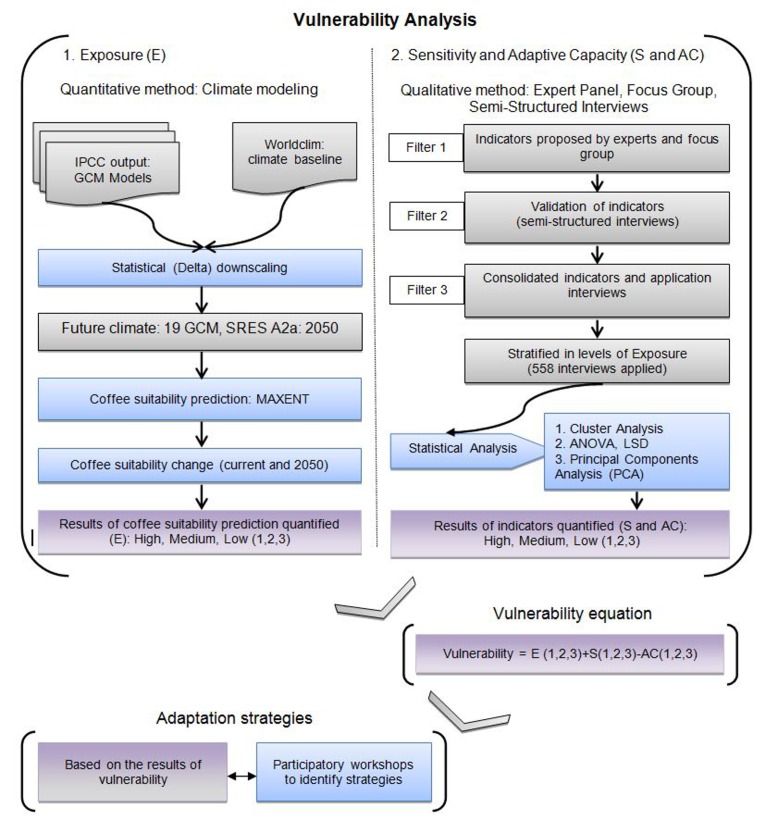
Framework to assess the vulnerability of coffee communities and to identify strategies for adaptation to climate change.

These factors are related to the interaction between climate change and access and availability of resources to farming families. Exposure is quantified by modelled coffee crop suitability change comparing current and future climates, representing how familieś livelihoods will be impacted by climate change. Sensitivity and adaptive capacity are measured by indicators based on family resources-such as-natural, human, social, physical and financial capital [1]. We quantified vulnerability levels by combining exposure, sensitivity and adaptive capacity. Then, we identified adaptation strategies based on vulnerability levels applying participatory methods with coffee producing communities and organizations (Figure 1). The communities and organizations included were from four countries. The study was part of a development project seeking to facilitate adaptation to climate change among coffee producers that was implemented in Mexico, Nicaragua, Guatemala and El Salvador. These countries represent a range of economic and social development in the region from Nicaragua the poorest to Mexico the richest country.

### Exposure

To quantify exposure to climate change, crop suitability models predicting future changes of climatic suitability of coffee were used for the four countries. The methodology combined current climate data with future climate change predictions. To map current climatic suitability, the historical climate database WorldClim (www.worldclim.org) was used. The variables included a total of 19 bioclimatic variables derived from monthly precipitation, monthly median temperature, minimum and maximum temperature [15]. Bioclimatic variables represent annual trends, seasonality, and extreme conditions.

To predict future climate, the SRES-A2a scenario 19 IPCC Global Circulation Models were used. The Delta method was used to down-scale the climate change data, based on the sum of the anomalies interpolated with the WorldClim monthly high-resolution surfaces [15]. The method produces a softened surface (interpolation) of climate change (deltas or anomalies). It implies that changes in climate are only relevant at coarse-scale and that the interactions between variables are maintained in the future [16].

The Maximum entropy (MAXENT) method, a general-purpose method for making predictions or inferences based on incomplete information [17], was used to predict the future climatic suitability for coffee. The model requires calibration with climate data for current coffee production areas, which is provided by GPS coordinates. The model assumes that a certain future climate at a given site is as suitable or unsuitable for the crop as is the same climate at another site in the present. This assumption is reasonable as long as crop genetics and cropping systems do not significantly change. It thus predicts what will happen in terms of relative climatic suitability for a crop if these factors do not change and helps identify those sites where adaptations in crops and cropping systems are necessary in order to avoid the consequences of a predicted decline in climatic suitability. This approach has previously been used for coffee [6], [18].

Two measures of uncertainty were calculated: (1) the agreement of calculated models as a percentage of models that predict changes in the same direction and (2) the coefficient of variation (CV) among models.

### Sensitivity and adaptive capacity

Indicators of the sensitivity to climate change and adaptive capacity were devised in collaboration with organizations and experts from the region using an expert panel, focus groups, and semi-structured interviews. For the expert panel, semi-structured individual interviews were conducted with 17 key informants of the coffee sector in Nicaragua, including technicians, farmers and researchers. It included questions about the most important factors affecting coffee production. Four focus groups were carried out in Nicaragua and three groups in each of the remaining countries (El Salvador, Guatemala and Mexico). Participants discussed and assessed the significance of climate change over time and identified key indicators for coffee livelihoods. The list of key indicators was structured according to the five community capitals (natural, human, social, physical and financial) of the Livelihoods Approach [1].

Parameters were then constructed to evaluate each indicator as shown in Table S1 in File S1. To quantify the parameters scales from 1 to 5 were applied or a binary scale of 0 and 1, depending on the nature of the parameter. The final values for each indicator were calculated by averaging all the parameters and then transformed to a 0-1 continuous variable scale, with 0 being low and 1 being high sensitivity and adaptive capacity. For example, access to and availability of water is an important natural resource for familieś livelihoods and coffee production. To measure the water access and availability indicator we considered the parameters source, distance, quality and quantity of water. Water availability, for example, is measured on a scale of 1 to 5, 1 being least sensitive and 5 being most sensitive. A value of 1 means there is never sufficient water and a value of 5 means there is an abundance of water all year. Then we developed a semi-structured interview by adapting qualitative tools [19] and validated the tool with six coffee families.

The indicators were used to assess the vulnerability of coffee farms in each country. From a population of 7,000 farmer members from 15 organizations across the four countries, 558 farmers were interviewed. The farmers may be considered representative of small-scale organized farmers, but should not be considered representative of the coffee farmers as a whole in each country. The sample size was defined using the formula for finite populations [20] and then individual farmers were selected randomly, stratified according to exposure level and country by 2050 (Table 1).

**Table 1:** Number of interviewed families by country and exposure level^a^.

Country	Department or State		Exposure level		Total
		High	Medium	Low	
Nicaragua	Jinotega	12	14	15	41
	Matagalpa	36	20	5	61
	Madriz	4	14	14	32
	Nueva Segovia	0	0	16	16
	Total	50	50	50	150
El Salvador	Usulután	11	0	9	20
	Santa Ana	8	0	0	8
	La Libertad	4	11	14	29
	Ahuachapán	20	32	20	72
	Total	43	43	43	129
Guatemala	Chiquimula	14	23	10	47
	Sololá	17	2	4	23
	Chimaltenango	4	5	1	10
	San Marcos	8	13	28	49
	Total	43	43	43	129
México	Chiapas	4	0	32	36
	Oaxaca	45	30	11	86
	San Luis Potosí	1	20	7	28
	Total	50	50	50	150

a For the definition of exposure levels see section 2.3.

### Vulnerability

For exposure, the relative decreases in climatic suitability according to the MAXENT model were divided into three classes of suitability loss (low, medium, high). For sensitivity and adaptive capacity, indicators were identified and quantified through interviews with the farming families.

A cluster analysis was carried out for each indicator of sensitivity and adaptive capacity based on the score of each family using the Ward method with Euclidean distance. Then an Analysis of Variance (ANOVA) was applied using the LSD-Fisher test to compare the averages for each indicator by cluster. The indicators in each cluster that obtained significantly different sample averages were classified in three levels on a scale of 0 to 1 (0–0.33 = low, 0.34–0.66 = medium, 0.67–1 = high). Clusters with the greatest number of indicators with high, medium or low averages were classified as having high, medium or low sensitivity and adaptive capacity [21].

Each factor (exposure, sensitivity and adaptive capacity), as previously explained, and was classified into three levels (high, medium, low). To calculate the vulnerability equation we assigned each level a quantitative value: low = 1, medium = 2, high = 3. With three factors and three levels per factor, we obtained 27 possible combinations. After applying the equation we obtained 7 values (–1,0,1,2,3,4,5), which we used to define low (–1,0), medium (1,2,3,) and high (4,5) levels of vulnerability (Figure 1). A Principal Components Analysis (PCA) was carried out to identify the indicators that most contribute to the sensitivity or adaptive capacity of families in different municipalities.

### Identifying adaptation strategies

Workshops were carried out to identify possible adaptation strategies. Participants were families of farmers, technicians, and presidents of different cooperatives. Workshops began by presenting the results of the vulnerability analysis by state, department, or municipality. After a general discussion, the following question was asked to groups: Given that the conditions for coffee production will change by 2050, what can be done to maintain the level of production? This was first discussed in the plenary and then in subgroups of 3 to 5 people, followed by the presentation of results. Each participant then noted the three most important ideas from the discussion on separate cards and assigned them to one of the five types of resources (natural, human, social, physical and financial) where he felt they most closely linked. Then subgroups were formed for each resource type to organize the ideas and build an outline of a climate change adaptation strategy. In this strategy, key actors, roles, resource availability, and time needed to implement the strategy were identified. The subgroups presented their results to the entire group and received feedback, until a general consensus was reached for each adaptation strategy.

## Results

### Exposure

According to the climate change models, total annual precipitation in all countries is predicted to decrease by 2050. Nicaragua, already the driest of the four countries, is predicted to have a 5% precipitation decrease by 2050. The mean annual temperature will increase by 2.3°C, while the maximum temperature will increase in all countries by between 2.2°C and 2.4°C (Table S2 in File S1). The most decisive climatic variables for the predicted decrease in climatic suitability for coffee were the increase in maximum mean temperature of the hottest month. Mexico is predicted to reach a mean maximum temperature of 36°C by 2050.

In interviews the producers confirmed the trend of the climate models (Table S2 in File S1). They mentioned changes in climate seasonality and predictability, including hotter and longer dry seasons (from three-four months to four-six months) and shorter and drier wet seasons. Also, they perceived an increase in extreme temperatures, drought and wind as well as changes in the intensity and distribution of precipitation. They also highlighted an increase in extreme weather events, such as cold periods, hail, drought, and hurricanes.

The climatic suitability for Arabica coffee has been predicted to decrease in the lowest altitude areas as a result of the increase in temperature to which coffee quality is sensitive [22]. Changes in temperature and rainfall will decrease the area suitable for coffee and effectively displace coffee up the altitudinal gradient to cooler climates. On a national level, El Salvador and Nicaragua have the highest percentage of land affected by decreases in suitability of 40% or greater (Table 2). Models predicted that, in Central America as a whole, the optimal coffee-growing elevation will shift from 1,200 m.a.s.l currently to 1,600 m.a.s.l by 2050 [6], [23].

**Table 2:** Projected changes in overall suitability for coffee production and altitudinal range suitable for production in Mesoamerica by 2050.

Country		Changes in overall suitability for coffee production			Altitude suitable for production in meters above sea level	
	–40% or more	–40% to –20%	–20% to 0%	<0%	Current model	Future model
					(1950–2005)	(2050)
El Salvador	45.5	43.7	10.9	0	700 to 1700	1000 to 1700
Guatemala	12.9	25.5	54.2	7.4	600 to 1800	1200 to 2200
Mexico	18.2	34.6	46.9	0.3	500 to 2000	1200 to 2300
Nicaragua	35.3	32.1	32.5	0.1	700 to 1500	1000 to 1600

Adapted from Läderach et al. (2010b).

**Figure 2:**
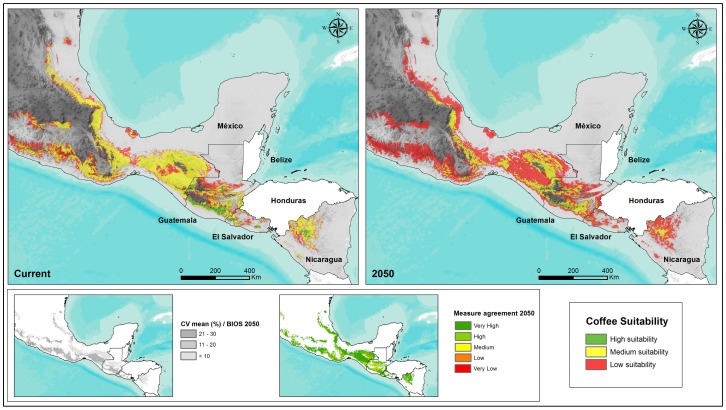
Prediction of the relative climatic suitability for Arabica coffee production in Mexico, Guatemala, El Salvador and Nicaragua in 2010 and 2050 (large maps), coefficient of variation (CV; small map to the left), and consistency between models (small map to the mid-right).

In general, the climatic suitability for Arabica coffee will be maintained at the highest altitudes. In Nicaragua, the area with the greatest loss in suitability is located in the Pacific zone in the departments of Carazo and Managua, while the highlands around Apanás Lake in Jinotega will maintain a high suitability for coffee quality. In El Salvador, the area with loss of suitability will be the departments of San Miguel and Usulután, while the department of Ahuachapán will maintain high suitability. In Guatemala, greatest loss of suitability will be the southern slope of the Pacific volcanic chain, northern Zacapa and eastern Chiquimula, while Chimaltenango will maintain high suitability. In Mexico, areas with greatest loss of suitability will be northern Chiapas, Veracruz and Tabasco, whereas high suitability will be maintained in the Chiapas highlands (Figure 2).

### Sensitivity and adaptive capacity

#### Sensitivity

The sampled families from each country were divided into three sensitivity levels using cluster analysis; on average 33% of the families fell into the highly sensitive level. The families of El Salvador and Guatemala were more likely to cluster in the high sensitivity level, while those of Nicaragua and Mexico were more likely to cluster in the medium sensitivity class. Guatemala had the highest percentage of families with high sensitivity (49%) and El Salvador the highest percentage with low sensitivity (34%) (Table 3).

**Table 3:** Distribution of families per level sensitivity between countries.

Country	High sensitivity (%)	Medium sensitivity (%)	Low sensitivity (%)	Total (%)
Nicaragua	22	61	17	100
El Salvador	40	26	34	100
Guatemala	49	37	14	100
Mexico	23	46	31	100

**Figure 3:**
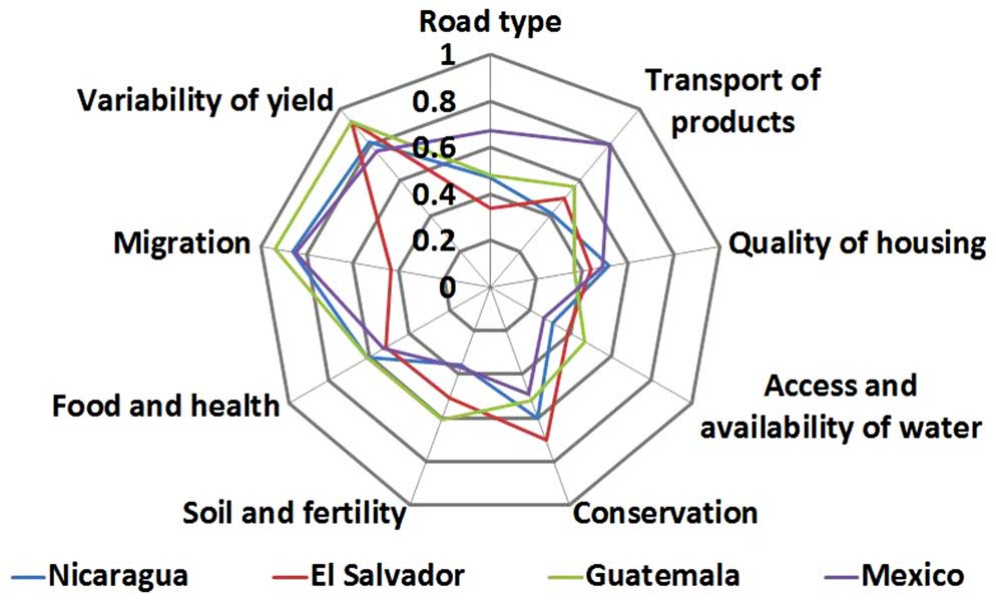
Sensitivity indicators in the livelihoods of small coffee producers to climate change in four countries of Mesoamerica (a high value equals high sensitivity).

An ANOVA test indicated that there are significant differences (p<0.001) between high, medium and low clusters for the sensitivity indicators for each country. Figure 3 shows some indicators that were significantly different in the high sensitivity level compared by cluster for each country. The families in the high sensitivity level in each country were characterized by high yield variability. In El Salvador, Guatemala and Nicaragua this indicator was significantly higher for high sensitivity than for medium or low sensitivity clusters (p<0.001). In contrast, in Mexico the indicator was not significantly different (p = 0.090) between sensitivity clusters (Figure 3). Yield variability leads to frequent reductions in income and the ability of the families to respond to external stresses such as climate change. Furthermore, in Nicaragua, Mexico and Guatemala the migration indicator was also significantly greater for the high sensitivity cluster in each country (p<0.001). Migration, being the temporary or permanent work-related move of one or more family members to a foreign country, reduces the availability of family labour and, thus, the resilience to respond to climate change impacts. The resulting lack of labour for the coffee harvest was frequently mentioned during the interviews.

In El Salvador, a notable contributor to high sensitivity was lack of conservation practices (p<0.001), including lack of maintenance of vegetation protecting water sources and communally or individually managed forest areas. In parts of the country, farm sizes are small (between 1–2 hectares) and therefore there is little space for leaving areas of natural vegetation. On the other hand, producers in some communities in the departments of Ahuachapán and La Libertad in El Salvador are organized in groups of 40–50 families that collectively own 600–1100 hectares of land on which they maintain water sources and forest areas. In Mexico, high sensitivity was particularly related to difficulties with transportation of products (p<0.001) (Figure 3).

**Figure 4:**
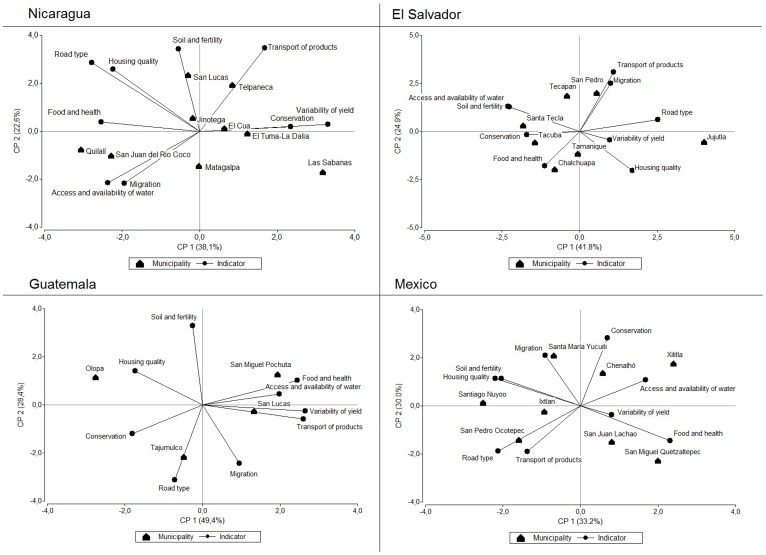
Principal components analysis of association of sensitivity indicators with different municipalities.

The Principal Components Analysis showed that key indicators of vulnerability differed among municipalities even within the same country. The value of the first axis and second axis per country is shown in the Figure 4. The variables that were highly correlated with axis 1 included yield variability (r = 0.48) in Nicaragua, road type (r = 0.48) in El Salvador, transport of products (r = 0.46) in Guatemala and, health and nutrition (r = 0.46) in Mexico. For example, poor transport and bus connections restricting market access was a key factor of vulnerability in San Lucas Tolimán in Sololá, Guatemala. Farmers grow coffee among the volcanoes surrounding Lake Atitlan and had to walk between 3–4 hours from their farms to the nearest market town, transporting their produce either on mules or on their backs. Similar conditions prevail in many coffee communities in remote mountain areas across the region.

In El Tuma-La Dalia in Matagalpa the coffee yield of producer farms had high variability between years. Ranges in yield were between +/– 25% on average for four years, with a variability of +/– 50% reported in some cases; the average farmer yield was 331 Kg/ha, in contrast with the Matagalpan department average of 538 Kg/ha. High variability of yields and thus income between years limited the access and availability of food, nutrition, education and health of families. Sometimes when the yield was very low, in Nicaragua the families migrate to other places in search of casual labour. Depending on the municipality or community, migration was an important factor in each country and included both seasonal and permanent migrants (e.g. in Matagalpa, Nicaragua, ten out of eleven interviewed families had some of its members living abroad as migrants) (Figure 4).

#### Adaptive capacity

The sampled families from each of the four countries were divided into three adaptive capacity levels using the cluster analysis; on average, 32% of families fell into the low adaptive capacity level. The families of Nicaragua, El Salvador and Guatemala were more likely to cluster in the low adaptive capacity level (Table 4).

**Table 4:** Distribution of families per level adaptive capacity between countries.

Country	High adaptive capacity (%)	Medium adaptive capacity (%)	Low adaptive capacity (%)	Total (%)
Nicaragua	41	22	37	100
El Salvador	50	15	35	100
Guatemala	53	13	34	100
Mexico	38	38	24	100

**Figure 5:**
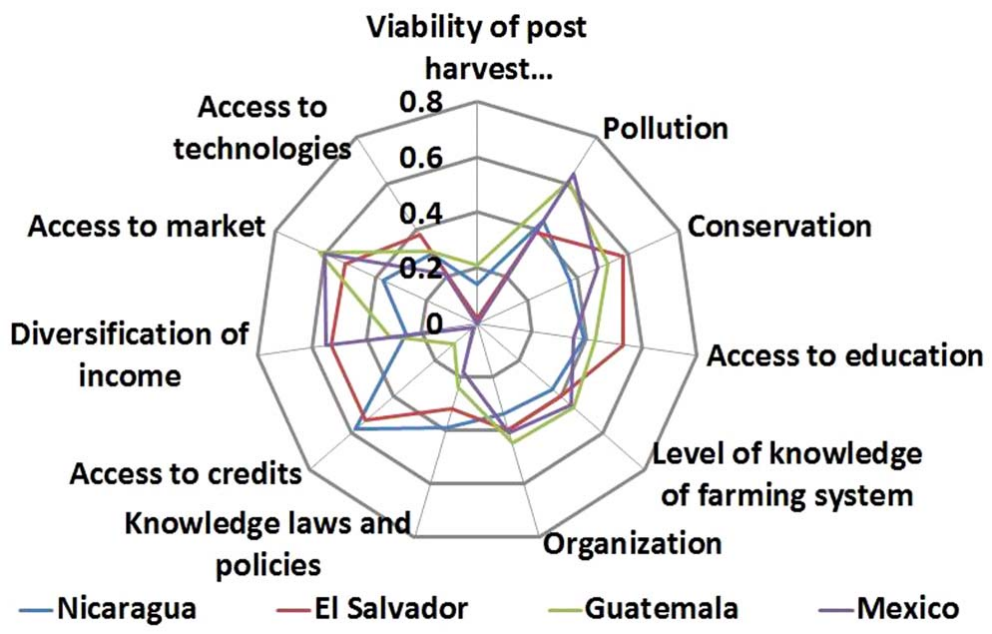
Adaptive capacity indicators in the livelihoods of small coffee producers to climate change in four countries of Mesoamerica (a low value equals low adaptive capacity).

The ANOVA test indicated significant differences between the high, medium and low adaptive capacity clusters in each country, but the indicators varied across countries. Figure 5 shows indicators that were significantly different in low adaptive capacity level compared to clusters for each country. The families in the low adaptive capacity cluster, in Mexico, Guatemala, El Salvador and Nicaragua were characterized by low viability of post harvest infrastructure for drying coffee. Low adaptive capacity families in Mexico and El Salvador had poorer post harvest infrastructure for drying coffee (p<0.001), than higher adaptive capacity families. Between 50 and 80% of families interviewed had only a single technique for drying coffee irrespective of weather conditions. While in Mexico, El Salvador and Guatemala producers had access to drying patios, in Nicaragua producers dry their coffee using drying tables, drying patios or plastic tarps and, in Mexico producer dry coffee using sacks, or on the floors or house roofs. In very humid regions, these drying techniques were not suitable due to lack of adequate sun; as a result, the drying quality is poor. Some cooperatives in Nicaragua and Guatemala with economic resources transport the coffee to drier areas, while other cooperatives use a mechanical drying process. In both cases this increases the cost of processing.

Furthermore, low adaptive capacity farmers in Nicaragua presented lower income diversification (p<0.005).Families depended on coffee sales for between 50 to 60% of their yearly income. Some families diversify their income with basic grains, bananas, oranges and avocados, but the intermediaries pay very little and the markets are distant. In all countries farmers received credit from cooperatives. However, in Guatemala low adaptive capacity farmers had more limited access to credit (p<0.001) than higher adaptive capacity farmers, with 27% of 129 farmers interviewed having had no access to credits. In Mexico low adaptive capacity farmers had lower knowledge levels regarding coffee sector policies and environmental and land use laws (p<0.001), than higher adaptive capacity famers. In general, interviewed families had knowledge of only one to three coffee sector or environmental policies, and they did not have active participation in the application of these laws. Additionally, in Mexico low adaptive capacity families had low access to alternative technologies, but this result was not significantly different from the other adaptive capacity levels (p = 0.079). For example, they did not collect water for their own consumption or for crops, and they did not use drip irrigation (Figure 5).

**Figure 6:**
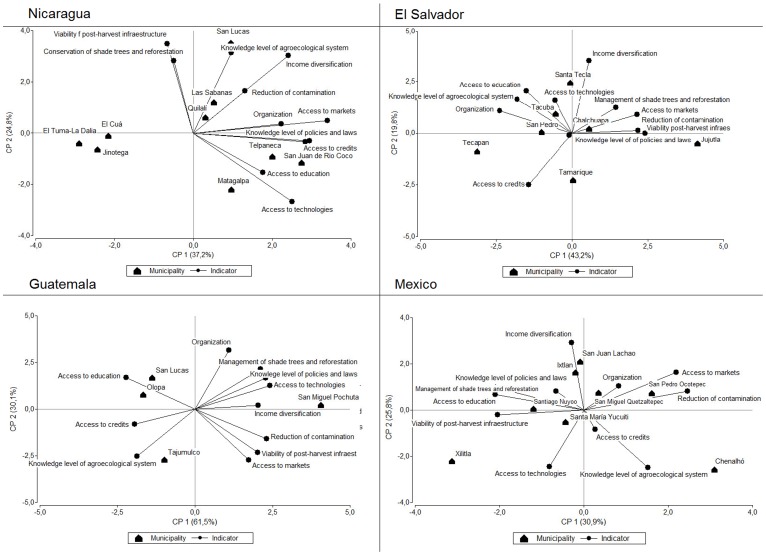
Principal components analysis of the association of adaptive capacity indicators to different municipalities.

There was a close association of some municipalities with certain indicators in the Principal Components Analysis. The value of the first axis and second axis per country is shown in Figure 6. The variables that were least correlated with axis 1 included post harvest infrastructure viability (r = –0.09) in Nicaragua and (r = –0.159) Mexico, organization (r = –0.43) in El Salvador and, education (r = –0.31) in Guatemala. For example, in Xilitla in San Luis de Potosí, Mexico interviewed families had little access to post-harvest infrastructure; farmers dry their coffee on the floor, in sacks, on plastic or other forms, and some producers sell their coffee as unprocessed cherries with corresponding price reductions (Figure 6).

In San Lucas Tolimán in Sololá, Guatemala, the families had little access to formal and informal education. This is because the heads of household are of Mayan origin and speak their local language, rather than Spanish. They had little access to education, and there is no technical assistance in their own language. Currently children have access to primary school and Spanish language instruction.

### Vulnerability and adaptation strategies

The results of the vulnerability equation indicate that in Nicaragua, of the 143 families, 18% had a high level of vulnerability; in El Salvador, 14% out of 129 interviewed families showed high vulnerability; in Guatemala, 22% out of 129 families showed high vulnerability; and in Mexico 9% out of 150 families showed high vulnerability (Table 5).

**Table 5:** Percentage of families by vulnerability level in each country.

Country	Department or state	Vulnerability level	Total (%)
		High	Medium	Low	
Nicaragua	Jinotega	6	13	10	29
	Matagalpa	9	22	6	38
	Madriz	3	10	9	22
	Nueva Segovia	0	6	5	11
	Total (%)	18	51	31	100
El Salvador	Usulután	5	10	1	16
	Santa Ana	2	5	0	6
	La Libertad	5	10	8	22
	Ahuachapán	3	43	9	56
	Total (%)	14	68	18	100
Guatemala	Chiquimula	13	22	2	36
	Sololá	9	9	0	18
	Chimaltenango	0	7	1	8
	San Marcos	0	26	12	38
	Total (%)	22	64	15	100
México	Chiapas	1	15	9	24
	Oaxaca	9	40	9	57
	San Luis Potosí	0	18	1	19
	Total (%)	9	73	18	100

**Figure 7:**
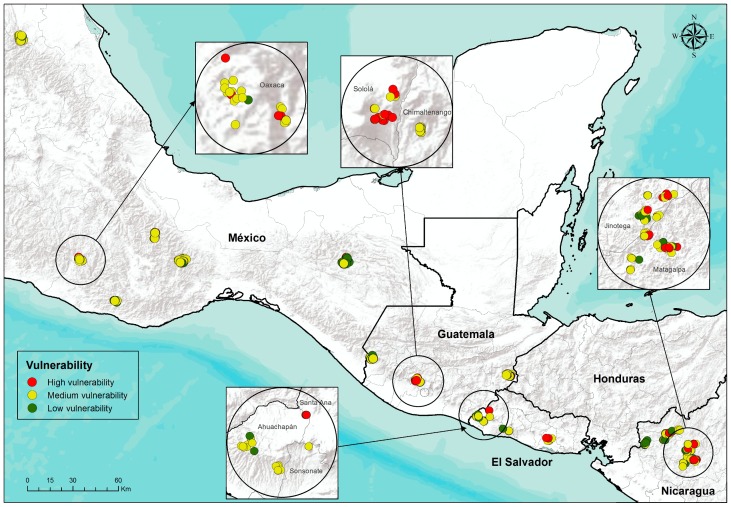
Small-scale variability of vulnerability to climate change among coffee producing communities in four countries of Mesoamerica.

High vulnerability was related to high and medium exposure, which was represented by a loss of climatic suitability for coffee production but in Mexico this tended to be less severe than in the three countries further south. The families’ farms located in the high exposure level will not have optimal conditions for production quality coffee by 2050 (Figure 7).

In addition high vulnerability was related to high and medium sensitivity, which was due to the high variability in productivity levels. The variability of production is very important for the families because it represents their principal income (on average 50 to 65%) and they depend on this income for food security, health and education. Furthermore, Nicaragua, Guatemala and Mexico had high sensitivity caused by migration. Additionally, high vulnerability related to low adaptive capacity resulted in all four countries from poor post-harvest infrastructure and limited access to credit and alternative technologies. Often the individual producers did not have access to machines for pulping coffee, drying infrastructure, solar dryers, drip irrigation or water harvesting. The low level of organization was due to the lack of participation of many families in joint activities, projects, training and exchanges. The low level of income diversification was because many families depend mainly on coffee for cash to buy food, healthcare, education and transportation or to invest in the farms (Table 6).

**Table 6:** Vulnerability indicators in relation to adaptation strategies and their specific adaption options.

Vulnerability indicators	Adaptation strategies	Specific adaptations options
Decrease of suitability for coffee production	Programs of research, validation, transfer and adoption of agricultural technologies that adapt coffee to changing climate	Drip irrigation, water harvesting and management of available water
High variability of annual productivity		Management of shade, fertility, crop residues, pest and diseases
Low soil fertility and forest conservation		Conservation of soil, water and natural forest
Low income diversification		Improved varieties and hybrids
		Diversification with other crops where loss of suitability for coffee production
Poor health and nutrition	Integral programs with Institutional support improving human and social resources	Improved environmental education (schools, organizations, committees)
Low level of organizational capacity		Implementing food and health security programs
Low level of knowledge of polices of coffee sector and local laws		Provide cooperatives with social experts to improve the level of participation of producers
High migration rate		Empowering families in policies and laws of their environment sector and to improve implementation
Low access to credit	Implementation of long term financial rural programs	Financial education
Low viability of post harvest infrastructure		Planning for the investment of resources
Low access to technologies		Planning of long-term credits (in cash, tools, supplies and others) with technical assistance
Low access to transport and types of roads	Implementation of investment programs to improve road infrastructure, quality housing and basic services	Planning with municipalities, private sector, international cooperation

Families identified as general strategies for adaptation to climate change the need to develop or improve technologies such as drip irrigation in areas with high risk of drought, shade management, soil fertility management, pest and disease control, conservation of soil and ground water, and adoption of new crops to adapt to future conditions.

## Discussion

The strategy used to determine and integrate estimates of exposure, sensitivity and capacity to adapt to climate change proved effective to differentiate between high, medium and low vulnerability families and to identify the livelihood characteristics that contribute to those states. Nevertheless, factors that contribute to vulnerability are distinct between departments, municipalities and families. This is consistent with Cutteŕs analysis that “it is place that forms the fundamental unit of analysis” for vulnerability [24]. Thus it is not unexpected that the nature of vulnerability is very site or even family specific [25].

The farms located in areas with high vulnerability level will not have suitable conditions for quality coffee production by 2050. These conditions include changing climate factors (e.g. temperature, precipitation) by 2050, high variability of coffee production and high levels of migration in some communities, low adaptive capacity in post harvest infrastructure and in Guatemala and Mexico low access to credit. Some of these are similar to those identified by Eakin [26] as the important drivers of change, which include: torrential rainfall, credit, declining soil fertility, new market opportunities, and declining international coffee price. Tucker found that coffee farmers in Central America primarily adapted to global change through changes in crops and crop practices, supporting the importance of diversification options. Nevertheless, these changes were not associated with perceptions of climate risk but rather with market demands [10]. We found that some communities in Mexico, Nicaragua and Guatemala had high migration rates as an additional characteristic of high vulnerability. While migration is definitely on the rise in many parts of Mesoamerica, it appeared that migration in coffee households was difficult to link specifically to climate change drivers [26].

In areas that will remain suitable for coffee growing, but with some reductions in suitability, better agronomic management could lessen the impacts of climate change [6], while in those where a low suitability for coffee growing has been predicted, farmers will have to identify crop alternatives. Solving the problem of variable yield is crucial to the survival of farmers who live in marginalized environments where agro-climatic conditions have always been a challenge. Diversification is, therefore, an important strategy for production risk management in small farming systems. In general, traditional agro-ecosystems are less vulnerable to catastrophic loss because the wide variety of crops and various spatial and temporal arrangements show compensation in case of loss [27].

The adaptation of smallholders also relates to the global supply chain, which is governed by traders and industries that determine the market price and requirements, considering that an upgrading of the coffee chain could help reduce its economic vulnerability [28]. But ultimately it is the farmers who decide what to farm and how. Strategic decisions must be made with the uncertainty of climate conditions, pricing, costs, government programs, and others factors [29].

Adaptation solutions should focus on the development of new infrastructure, policies, and support institutions that facilitate, coordinate, and maximize the benefits of the new systems of land management and use. This can be accomplished by improving governance, ensuring that development programs take climate change into account; increasing investments in irrigation infrastructure and technologies that would increase water use efficiency; creating appropriate transportation and storage infrastructure; reviewing the agrarian property regime and establishing accessible, efficient markets for products, assets and financial services including insurance [30].Finance is critical: access to microloans and formal credit for farm-level investments will help households strategically invest in coffee varieties, complementary crops and livelihood enhancements that effectively reduce risk and improve social welfare [26].

Nevertheless, the solutions require the support of social organizations (civil society groups, cooperatives and small-business organizations) to enable rural households to access the resources and knowledge necessary for adaptation, while empowering communities to shape the direction of the coffee sector to meet their diverse development needs [26]. In the case of coping with weather-related hazards, social networks play a primary role in adaptation and recovery. Social and institutional diversity itself promotes resilience [25]. The decision of a farmer to participate in such an organization is thought to provide improved access to resources and knowledge, as well as to provide a social network that could facilitate recovery and re-establishment of activities following severe shocks (e.g. climatic and price changes). Facilitating adaptation will involve renewed attention to helping households acquire information about markets and new technology. In the face of declining public investment in agriculture, it is clear that public support is needed for research on low-cost, low-input strategies to manage climatic extremes [26]. Nevertheless, investment is also needed in the social organization to enable the adaptation of these strategies to local community and individual family vulnerability characteristics.

The role of the state remains important for planned adaptation and sustainable development. Governance is vital in managing global environmental risks and in promoting sustainable technologies. Also, it is necessary for the implementation of human and social programs to have government support and participation of organizations, social networks, education centres and the international cooperation [31].

## Supporting Information

File S1 **Table S1, Key sustainable livelihood indicators of sensitivity and adaptive capacity of coffee producing families in Mesoamerica. Table S2. Current and future values of bioclimatic variables with (averages of coffee growing area in each country) their coefficients of variation (CV) of 19 GCM models for Nicaragua, El Salvador, Guatemala and Mexico.** The CV indicates the variation between models (i.e. the greater the CV the more variability between GCMs).
(DOCX)
