# Supplementary material for: An Integrated Framework for Assessing Vulnerability to Climate Change and Developing Adaptation Strategies for Coffee Growing Families in Mesoamerica
Source: PLoS One. 2014 Feb 26;9(2):e88463. doi: 10.1371/journal.pone.0088463 (PMC3935832; doi:10.1371/journal.pone.0088463)
Supplement: File S1 — Table S1, Key sustainable livelihood indicators of sensitivity and adaptive capacity of coffee producing families in Mesoamerica. Table S2. Current and future values of bioclimatic variables with (averages of coffee growing area in each country) their coefficients of variation (CV) of 19 GCM models for Nicaragua, El Salvador, Guatemala and Mexico. The CV indicates the variation between models (i.e. the greater the CV the more variability between GCMs). (DOCX) [file pone.0088463.s001.docx]

**Supplementary materials**

Table S1: Key sustainable livelihood indicators of sensitivity and adaptive capacity of coffee producing families in Mesoamerica

| **Vulnerability** | **Capital** | **Indicator** | **Parameter** | **Measure** |
| --- | --- | --- | --- | --- |
| Sensitivity | Natural | Conservation | Area of forest around the water source | Area in hectare |
|  |  |  | Area of forest to keep in the farm | Area in hectare |
|  |  | Soil and fertility | Soil type | Loam, sand or clay |
|  |  |  | Soil slope | Flat, medium or steep slope |
|  |  |  | Mulch of leaves | Coverage in percent |
|  |  |  | Soil depth | Depth in centimeters |
|  |  | Access to and availability of water | Source of water for drinking or postharvest processing | Origin of water |
|  |  |  | Availability of water during the year | Time of availability in the year |
|  |  |  | Distance to the water source | Distance in meter or kilometer |
|  |  |  | Water quality | Perception of quality (good, medium, bad) |
|  | Financial | Variability of annual coffee production | Average farm yield in four years compared to the local average | Weighted average in percent |
|  | Physical | Road type | Time from the farm to the collection center | Time in hours |
|  |  |  | Time from the farm to the nearest market | Time in hours |
|  |  |  | Type of road from the farm to the collection center or nearest market | Type of road (earth, paved, gravel) |
|  |  | Transport of products | Type of transportation from the farm to the market | Form of transport (men, bus, others) |
|  |  |  | Time from the farm to the bus stop | Time in hours |
|  |  | Housing quality | Housing material  Basic services | Type of material (bricks, wood, others)  Number of services available |
|  |  |  |  |  |
|  | Human | Health and food | Number of symptoms of human disease | Number |
|  |  |  | Number of times that person is attended by a doctor | Number |
|  |  |  | Dependency of external products | Percent |
|  |  | Migration | Type and time | Permanent/temporary and year/month |
| Adaptive capacity | Natural | Management of shade trees and reforestation | Number of trees cut | Number |
|  |  |  | Number of trees planted | Number |
|  |  | Pollution | Waste management | Burning, recycle, compost |
|  |  |  | Release of fermentation residues into water | Nothing, recycle, precipitated, filtered |
|  |  |  | Management of agrochemical containers | Nothing, builds, apply fresh compost |
|  |  |  | Coffee waste management | Nothing, recycle, burn, bury, return to field |
|  |  |  | Area burning annually | Hectares |
|  | Physical | Viability of post-harvest infra. | Types or forms to dry coffee | Number |
|  | Financial | Access to credits | Term of credit | Time |
|  |  |  | Interest rate of credit | Percent |
|  |  |  | Opportunity of credits | Time |
|  |  | Income diversification | Number of sources of income | Number |
|  |  | Access to specialty markets | Destined for sale | Percentage |
|  |  |  | Special market access | Number of certifications |
|  |  | Access to alternative technologies | Varieties | Knowledge and availability |
|  |  |  | Drip irrigation | Access and availability |
|  |  |  | Water harvesting | Access and availability |
|  | Social | Organization | Participation | Number of organizations |
|  |  |  | Time | Years |
|  |  |  | Benefits | Number |
|  |  | Knowledge level of policies related to the coffee sector, environmental laws and other | Policies about coffee sector | Knowledge and application |
|  |  |  | Environmental laws | Knowledge and application |
|  |  |  | Land polices | Knowledge and application |
|  | Human | Access to formal and informal education | Level of education | Non, primary, secondary, higher |
|  |  |  | Quality of technical assistance | Perception (Good, medium, poor) |
|  |  |  | Crops for which receive technical assistance | Number |
|  |  |  | Types of media accessed | Number |
|  |  | Knowledge level of agro ecological system | Registration practices and activities | Number of practices registered |
|  |  |  | Coffee intercropping | Number of types of crop |
|  |  |  | Pests and diseases | Percentage (Incidence and severity) |

Table S2: Current and future values of bioclimatic variables with (averages of coffee growing area in each country) their coefficients of variation (CV) of 19 GCM models for Nicaragua, El Salvador, Guatemala and Mexico. The CV indicates the variation between models (i.e. the greater the CV the more variability between GCMs).

| Variable | Description | Nicaragua | | | El Salvador | | | Guatemala | | | Mexico | | |
| --- | --- | --- | --- | --- | --- | --- | --- | --- | --- | --- | --- | --- | --- |
|  |  | Current | 2050 | | Current | 2050 | | Current | 2050 | | Current | 2050 | |
|  |  | Mean | CV% | Mean | Mean | CV% | Mean | Mean | CV% | Mean | Mean | CV% | Mean |
| Bio 1 | Annual mean temperature (^0^C) | 22 | 3 | 24 | 22 | 3 | 24 | 21 | 3 | 23 | 22 | 3 | 25 |
| Bio 2 | Mean diurnal range (Mean of monthly (max temp-min temp)) (^0^C) | 10 | 17 | 11 | 12 | 13 | 12 | 11 | 16 | 12 | 13 | 16 | 14 |
| Bio 3 | Isothermality (Bio2/Bio7) (*100) (^0^C) | 7 | 3 | 7 | 8 | 3 | 7 | 7 | 3 | 7 | 6 | 5 | 6 |
| Bio 4 | Temperature seasonality (Standard deviation*100) | 95 | 15 | 108 | 76 | 15 | 89 | 86 | 16 | 106 | 275 | 9 | 299 |
| Bio 5 | Maximum temperature of warmest month (^0^C) | 29 | 6 | 31 | 30 | 5 | 32 | 28 | 6 | 31 | 33 | 7 | 36 |
| Bio 6 | Minimum temperature of coldest month (^0^C) | 14 | 4 | 16 | 14 | 4 | 16 | 13 | 4 | 15 | 11 | 6 | 13 |
| Bio 7 | Temperature annual range (Bio5 GBi06) (^0^C) | 14 | 15 | 15 | 15 | 11 | 16 | 15 | 14 | 16 | 22 | 13 | 23 |
| Bio 8 | Mean temperature of wettest quarter (^0^C) | 22 | 4 | 24 | 21 | 3 | 24 | 21 | 3 | 23 | 25 | 3 | 27 |
| Bio 9 | Mean temperature of driest quarter (^0^C) | 21 | 4 | 24 | 21 | 3 | 23 | 20 | 4 | 22 | 19 | 5 | 22 |
| Bio 10 | Mean temperature of warmest quarter (^0^C) | 23 | 4 | 25 | 23 | 3 | 25 | 22 | 3 | 24 | 25 | 4 | 28 |
| Bio 11 | Mean temperature of coldest quarter (^0^C) | 20 | 3 | 22 | 21 | 3 | 23 | 20 | 3 | 22 | 19 | 4 | 21 |
| Bio 12 | Annual precipitation (mm) | 1832 | 10 | 1735 | 2008 | 8 | 1945 | 2340 | 9 | 2290 | 2114 | 6 | 2025 |
| Bio 13 | Precipitation of wettest month (mm) | 296 | 7 | 293 | 417 | 9 | 415 | 437 | 11 | 444 | 435 | 7 | 432 |
| Bio 14 | Precipitation off driest month (mm) | 26 | 17 | 24 | 3 | 66 | 2 | 23 | 40 | 20 | 49 | 31 | 36 |
| Bio 15 | Precipitation seasonality (Coefficient of variation) | 65 | 4 | 66 | 93 | 3 | 94 | 81 | 4 | 83 | 74 | 6 | 78 |
| Bio 16 | Precipitation of wettest quarter (mm) | 799 | 9 | 765 | 1058 | 8 | 1041 | 1089 | 10 | 1092 | 1028 | 7 | 1004 |
| Bio 17 | Precipitation of driest quarter (mm) | 95 | 11 | 93 | 16 | 37 | 13 | 85 | 22 | 77 | 158 | 20 | 128 |
| Bio 18 | Precipitation of warmest quarter (mm) | 464 | 20 | 465 | 254 | 47 | 289 | 584 | 31 | 611 | 692 | 19 | 637 |
| Bio 19 | Precipitation of coldest quarter (mm) | 202 | 23 | 215 | 168 | 74 | 194 | 146 | 46 | 151 | 174 | 22 | 161 |
